# Machine learning applied to serum and cerebrospinal fluid metabolomes revealed altered arginine metabolism in neonatal sepsis with meningoencephalitis

**DOI:** 10.1016/j.csbj.2021.05.024

**Published:** 2021-05-18

**Authors:** Peng Zhang, Zhangxing Wang, Huixian Qiu, Wenhao Zhou, Mingbang Wang, Guoqiang Cheng

**Affiliations:** a Division of Neonatology, Children’s Hospital of Fudan University, National Center for Children's Health, Shanghai 201102, China; b Division of Neonatology, Shenzhen Longhua People’s Hospital, Guangdong 518109, China; c Division of Neonatology, Longgang Central Hospital of Shenzhen, Guangdong 518116, China; d Shanghai Key Laboratory of Birth Defects, Children’s Hospital of Fudan University, National Center for Children’s Health, Shanghai 201102, China; e State Key Laboratory of Medical Neurobiology and MOE Frontiers Center for Brain Science, Institutes of Brain Science, Fudan University, Shanghai, 201102, China

**Keywords:** Neonatal sepsis, Meningoencephalitis, Metabolomics, arginine, machine learning

## Abstract

**Background:**

Neonatal sepsis with meningoencephalitis is a common complication of sepsis, which is a leading cause of neonatal death and neurological dysfunction. Early identification of neonatal sepsis with meningoencephalitis is particularly important for reducing brain damage. We recruited 70 patients with neonatal sepsis, 42 of which were diagnosed as meningoencephalitis, and collected cerebrospinal fluid (CSF) and serum samples. The purpose of this study was to find neonatal sepsis with meningoencephalitis-related markers using unbiased metabolomics technology and artificial intelligence analysis based on machine learning methods.

**Results:**

We found that the characteristics of neonatal sepsis with meningoencephalitis were manifested mainly as significant decreases in the concentrations of homo-l-arginine, creatinine, and other arginine metabolites in serum and CSF, suggesting possible changes in nitric oxide synthesis. The antioxidants taurine and proline in the serum of the neonatal sepsis with meningoencephalitis increased significantly, suggesting abnormal oxidative stress. Potentially harmful bile salts and aromatic compounds were significantly increased in the serum of the group with meningoencephalitis. We compared different machine learning methods and found that the lasso algorithm performed best. Combining the lasso and XGBoost algorithms was successful in predicting the concentration of homo-l-arginine in CSF per the concentrations of metabolite markers in the serum.

**Conclusions:**

On the basis of machine learning combined with analysis of the serum and CSF metabolomes, we found metabolite markers related to neonatal sepsis with meningoencephalitis. The characteristics of neonatal sepsis with meningoencephalitis were manifested mainly by changes in arginine metabolism and related changes in creatinine metabolism.

## Graphical abstract

**Figure:**
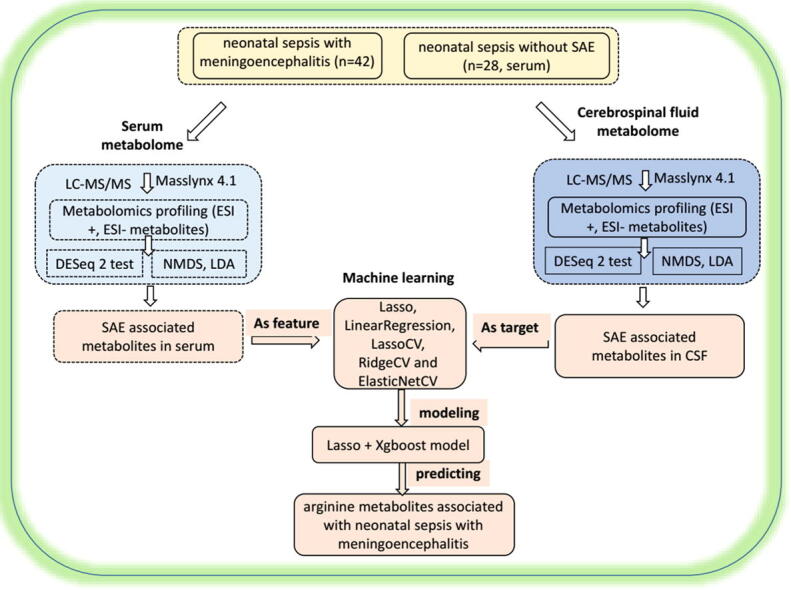

## 1 Introduction

Sepsis, a systemic inflammatory response syndrome caused by various pathogen infections, has high morbidity and mortality. Approximately 3 million newborns worldwide are diagnosed with sepsis every year, and neonatal sepsis is the most common cause of death in preterm and term infants [1], [2]. Meningoencephalitis（MEN） is a common complication of severe sepsis and patients with neonatal sepsis are prone to meningoencephalitis, which can lead to death and neurocognitive dysfunction. Sepsis-associated encephalopathyis defined as a diffuse brain dysfunction secondary to sepsis and without evidence of a primary CNS infection or encephalopathy due to other reasons [3]. Severe cases can be life-threatening and often cause brain damage or neurological sequelae, such as cerebral palsy, mental retardation, and deafness [4]. Therefore, early identification of neonatal sepsis with meningoencephalitis is particularly important for reducing brain damage.

Metabolomics technology is widely used to identify disease markers [5], and has been applied successfully to investigate sepsis markers [6], [7], [8], [9], [10], [11], [12]. Mickiewicz et al. [13] used nuclear magnetic resonance (NMR)-based targeted metabolomics and identified 186 metabolites in the serum of patients in a Pediatric Intensive Care Unit, suggesting that targeted metabolomics analysis may be a promising approach for the diagnosis and prediction of mortality in septic shock. Another NMR-based targeted metabolomics study [14] found that two metabolites (acylcarnitine C10:1 and glycerophospholipid PCaaC32:0) distinguished patients with severe sepsis from those with systemic inflammatory response syndrome. Using a targeted metabolomics approach, Fleischmann *et al*. found that a regression model based on two metabolites, sphingolipid SM C22:3 and glycerophospholipid lysoPCaC24:0, was able to diagnose sepsis with sensitivity of 84.1% and specificity of 85.7%. They also found that metabolites could effectively distinguish different infection types of sepsis and, thus, could be used as markers to predict patient prognosis. Liquid chromatography-tandem mass spectrometry (LC-MS/MS) combines high-performance liquid chromatography with electrospray ionization mass spectrometry metabolomics technology to achieve comprehensive detection of different types of metabolites in a sample, and has the advantages of high sensitivity and wide dynamic range over NMR-based targeted metabolomics technology [15].

Screening disease-related metabolite biomarkers and construction and optimization of diagnostic panels are the preliminary basis for the translation of laboratory research to clinical application research. However, mining metabolome data to discover disease biomarkers with high sensitivity, high robustness, and high accuracy still poses considerable challenges. In recent years, advanced machine learning algorithms have been widely used to screen medical biomarkers, and have performed well in finding disease-related metabolite markers [16], [17], [18]. The lasso (least absolute shrinkage and selection operator) algorithm is a machine learning method that simultaneously performs feature selection and regularization. Lasso can generate a refined linear model by constructing a penalty function, which also is an effective method to deal with complex collinearity data. Lasso has been successfully applied to metabolomics data analysis, and novel metabolite markers related to liver disease and neurological diseases have been found [19], [20], [21]. The aim of this study was to identify metabolites that distinguish the presence of meningoencephalitis in patients with neonatal sepsis from those septic patients without meningoencephalitis by performing LC-MS/MS metabolomics and applying the lasso algorithm.

## 2 Materials and methods

### 2.1 Participants

The present study was a retrospective collected analysis of the clinical data of patients with neonate sepsis, admitted to the Department of Neonatology at Children’s Hospital of Fudan University (Shanghai, China) from June 2019 to December 2019. Data were obtained from the medical files, and collected by a trained doctor. Excluded the following: patients who had hypoxia, pulmonary infection, urinary tract infection, congenital abnormality, cerebrovascular accident and pre-existing neurological syndromes. Diagnostic criteria for neonatal sepsis were, 1) positive blood culture and 2) non-specific signs and symptoms or focal signs of infection, and the detection of blood inflammatory response markers, specifically abnormalities in white blood cell count, C-reactive protein, procalcitonin, and/or platelet count [4]. Samples that had cerebrospinal fluid (CSF) puncture failure or contamination were excluded. Diagnostic criteria for neonatal sepsis with meningoencephalitis were, 1) positive for pathogens (bacteria or fungi) in CSF and 2) abnormal neurological symptoms, such as altered state of consciousness (irritability or unresponsiveness to stimulation), abnormal tone (hypo/hypertonia, abnormal posturing, decerebrate rigidity, or extensor response to painful stimulus), seizures, weak (or no) suck, and/or hypo/hyperventilation. Other patients with sepsis were considered controls, or septic patients without meningoencephalitis. This study was performed in accordance with the Declaration of Helsinki, and approved by the Ethics Committee of the Children’s Hospital of Fudan University. Informed written consent was obtained from a parent prior to study enrollment.

### 2.2 Sample collection

CSF (sterile tube, 0.5 ml) and peripheral venous blood (ethylenediaminetetraacetic acid tube, 2 ml) were collected simultaneously under sterile conditions. CSF and serum samples were collected immediately from each patient at the same time, the CSF and whole blood samples were centrifuged (1500 × *g* for 15 min) within 30 mins after collected. The resulting supernatant was dispensed and stored at − 80 °C until used.

### 2.3 LC-MS/MS

First, high-performance liquid chromatography separation was performed using an Ultimate 3000 LC system (Thermo Scientific, Waltham, MA, USA) coupled with an Acquity UPLC HSS T3 column (2.1 mm × 100 mm, 1.8 μm; Waters Corporation, Milford, MA, USA). Then, mass spectrometry was performed in both the positive and negative electrospray ionization modes (ESI + and ESI − ) using an Orbitrap Elite mass spectrometer (Thermo Scientific) following the manufacturer’s instructions, and as detailed in our previous studies [22], [23].

### 2.4 Metabolomics analysis

The Massynnx 4.1 software (Waters) was used to obtain the mass-to-charge ratio and peak intensity of each sample in the ESI + and ESI − modes. To assess whether the metabolomics data could distinguish patients with neonatal sepsis without meningoencephalitis from those with meningoencephalitis, we performed a sparse partial linear discriminant analysis using the ggord package in R (version 3.6.3) and a non-metric multidimensional scaling (NMDS) analysis using the vegan and ggplot2 packages in R. To identify specific metabolites that could distinguish patients with neonatal sepsis without meningoencephalitis from those with meningoencephalitis, we used the DESeq2 software [24] to screen out metabolites that were differentially abundant between the two groups. Metobolites with DESeq2.fdr ≤ 0.05 and |log2foldchange| ≥0.58 were considered to be significantly differentially abundant. Metabolite pathway enrichment analysis of the differentially abundant metabolites was performed using MetaboAnalyst 4.0 software [25] (http://metaboanalyst.ca).

### 2.5 Clinical correlation analysis

Linear relationship analysis between each metabolite and the clinical phenotype was performed using the lm function in R; p values < 0.05 were considered significant.

### 2.6 Receiver operating characteristic (ROC) analysis

To further determine whether the differential metabolites were useful markers for diagnosis of neonatal sepsis with meningoencephalitis, ROC analysis was performed for clinical inflammatory markers and significant metabolite markers using the pROC package in R. Area under the ROC curve (AUC) and the 95% confidence interval were obtained.

### 2.7 Machine learning models

**Data preprocessing**: Profiling the metabolite markers in serum and in CSF were selected as the feature and target, respectively. The log1p function in the NumPy library (version 1.18.5, abbreviated as np) was used to logarithmically transform the data. The train_test_split function in the scikit-learn (version 0.23.1) model_selection module was used to split the data into training and test datasets, with the parameters set as test_size = 0.2, random_state = 0.

**Linear model comparison**: We compared the prediction outcomes of four linear models, namely, linear regression, lasso regression, ridge regression, and elastic net regression. The scikit-learn linear_model module was used to import the LinearRegression, LassoCV, RidgeCV, and ElasticNetCV functions. First, per the training dataset, the GridSearchCV function of the scikit-learn model_selection module was used to find the optimal parameters for the four regression models; the parameters are poly__degree [3], [2], [1], poly__interaction_only [True, False], poly__include_bias [True, False], and linear__fit_intercept [True, False]. Then, the regression models, constructed on the basis of the optimal parameters, were used for data fitting, and the fitted models were used to predict the target value of the test dataset. Finally, the pyplot module of the matplotlib library (version 3.2.2) was used for data visualization.

**Lasso model prediction**: Lasso regression analysis was performed using the lasso function of the scikit-learn linear_model module. First, the parameters were tuned using the GridSearchCV function of the scikit-learn model_selection to find the optimal alpha parameter of the lasso regression model; alpha was set as [1e − 5, 1e − 4, 1e − 3, 1e − 2, 1, 5, 10, 20]. Then, the lasso regression model, constructed with the optimal parameters, was used to fit the training dataset. Finally, the fitted model was used to predict the target value of the test dataset, and the top 10 lasso regression coefficients were used for data visualization, which was done using the barh function of the matplotlib library (version 3.2.2).

**XGBoost model prediction**: XGBoost (eXtreme Gradient Boosting) regression analysis was performed using the XGBRegressor module of the XGBoost classifier (version 1.1.1). First, the XGBoost regression model was built with the parameters set as colsample_bytree = 0.3, gamma = 0.0, learning_rate = 0.01, max_depth = 4, min_child_weight = 1.5, n_estimators = 1668, reg_alpha = 1, reg_lambda = 0.6, subsample = 0.2, seed = 42, and silent = 1. Then, the built regression model was used to fit the training dataset. Finally, the fitted model was used to predict the target value of the test dataset.

**Lasso + XGBoost model prediction**: Because the performances of lasso and XGBoost were both good, we combined the two models for the regression analysis. Considering that lasso performed better than XGBoost on the training dataset, we set the weight of lasso as 0.6 and the weight of XGBoost lower as 0.4. The prediction value of the test dataset was (predictions_test) = np.expm1(0.6 × lasso_pred_test + 0.4 × y_pred_xgb_test), where np.expm1 is the inverse operation of the log1p function, lasso_pred_test is the lasso prediction value, and y_pred_xgost_test is the XGBoost prediction value.

## 3 Results

**Fig. 1:**
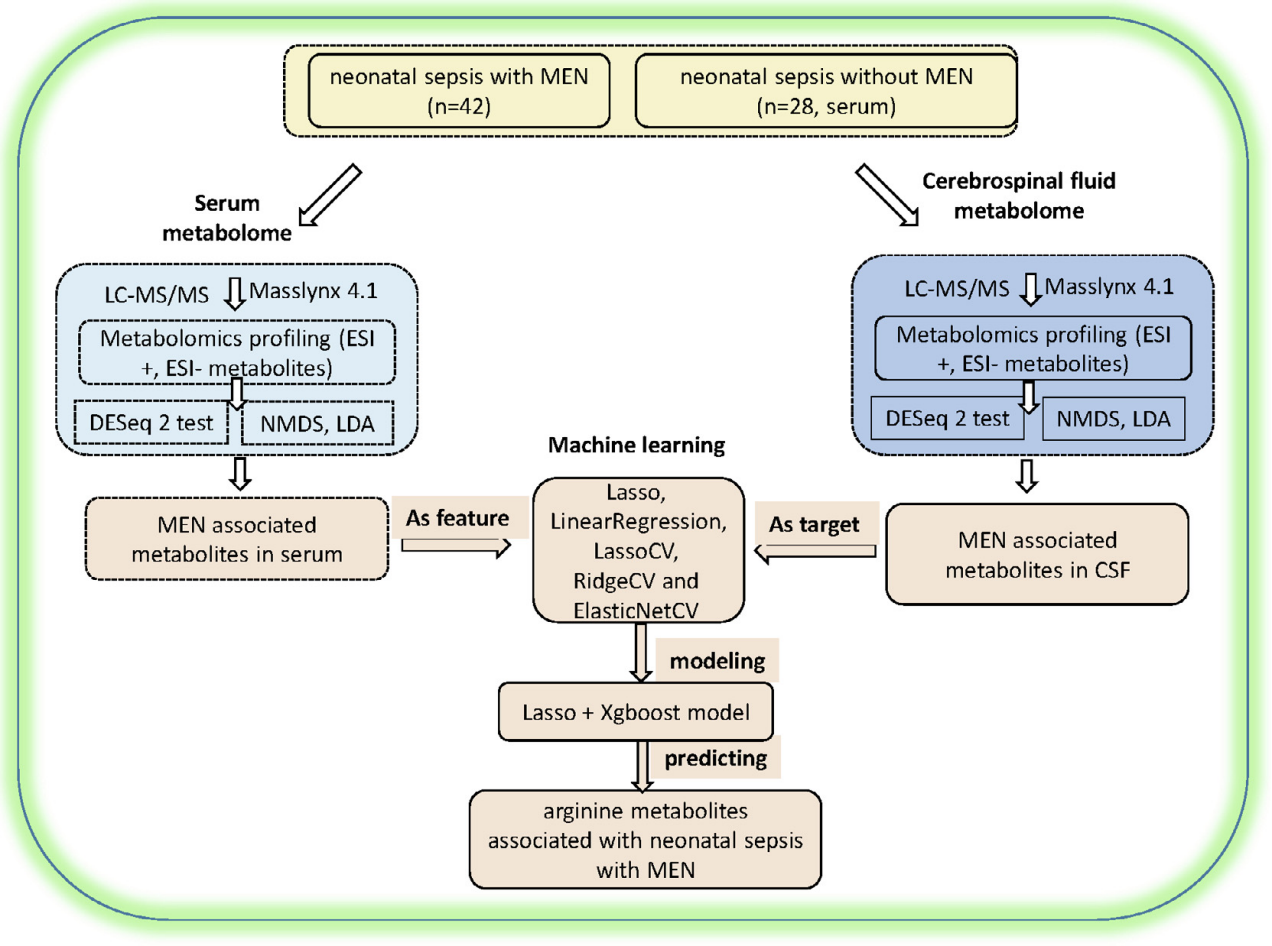
Flowchart of the study design. Forty-two patients with neonatal sepsis with meningoencephalitis and 28 patients with neonatal sepsis without meningoencephalitis were recruited. Cerebrospinal fluid (CSF) and serum samples were collected for LC-MS/MS detection, and metabolome-wide association analysis was performed to identify significantly different metabolites between neonatal sepsis with meningoencephalitis and without meningoencephalitis. Machine learning methods were used to predict the concentration of CSF metabolite markers per the determined concentration of these markers in the serum sample.

**Fig. 2:**
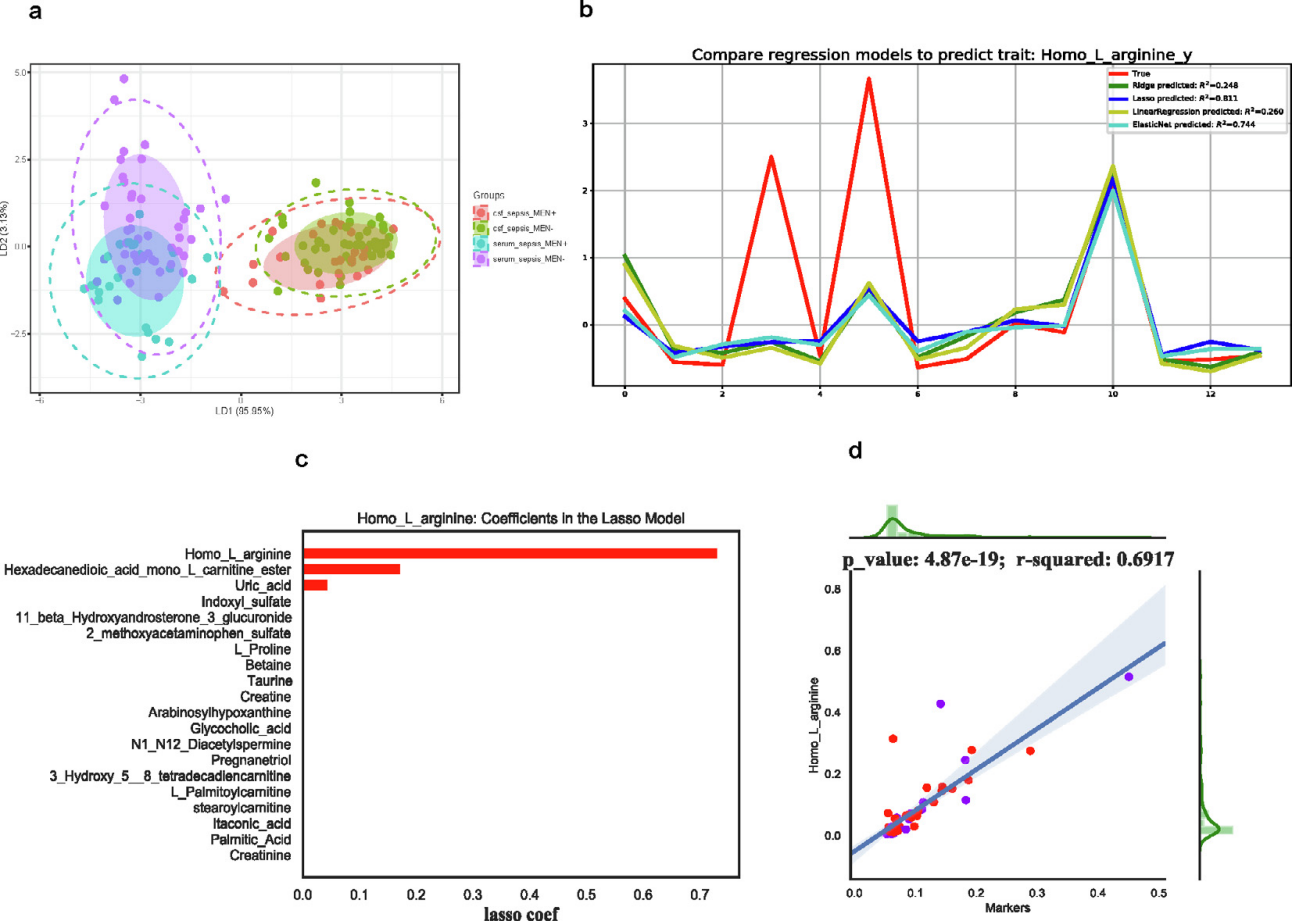
Altered homo-l-arginine levels in neonatal sepsis with meningoencephalitis. (a) Linear discriminant analysis (LDA) clearly distinguished the cerebrospinal fluid (CSF) and serum metabolomes in the LD1 dimension. (b) Performances of different machine learning models in predicting the homo-l-arginine concentration in the CSF on the basis of the metabolite marker concentrations in the serum sample. (c) Importance of the contribution of serum metabolite markers to the CSF metabolite concentration using the lasso model. (d) Predicted homo-l-arginine concentration in the CSF using lasso combined with XGBoost.

A flowchart of the study design is shown in Fig. 1. A total of 70 patients with neonatal sepsis were enrolled; 42 and 28 patients were with meningoencephalitis and without meningoencephalitis, respectively. The clinical data for these patients are summarized in Table 1. The CSF and serum samples collected from all 70 patients with neonatal sepsis were used in the LC-MS/MS-based metabolome assays. A total of 91 metabolites were detected in the CSF and serum samples by LC-MS/MS; 55 were detected in the ESI + mode and 36 were detected in the ESI − mode. The linear discriminant analysis showed that the metabolome of the CSF and serum samples was clearly distinguished in the LD1 dimension (Fig. 2**a**), indicating that the overall metabolomes of the CSF and serum samples were quite different. Furthermore, we also performed a NMDS analysis of the CSF and serum samples, which indicated that the CSF and serum metabolite data clearly distinguished patients with neonatal sepsis with meningoencephalitis from those without meningoencephalitis (Supplementary Fig. 1). This result is consistent with the results of the linear discriminant analysis.

**Table 1:** Clinical data for the patients with neonatal sepsis enrolled in this study.

	Neonatal sepsis with meningoencephalitis (n = 42)	Neonatal sepsis without MEN (n = 28)	*P* value
Serum samples (n = )	42, LC-MS/MS	28, LC-MS/MS	NA
CSF samples (n = )	42, LC-MS/MS	28, LC-MS/MS	NA
GA (weeks, mean ± SD[range])	35.76 ± 4.39 [26], [27], [28], [29], [30], [31], [32], [33], [34], [35], [36], [37], [38], [39], [40], [41]	38.3 ± 2.93 [29], [30], [31], [32], [33], [34], [35], [36], [37], [38], [39], [40], [41]	0.005671572
BW (g, mean ± SD[range])	2719.05 ± 1048.52[860–4260]	3223.93 ± 630.6[1365–4200]	0.014384012
Gender (n = female/male)	21/21	14/14	1
CRP (mg/l, mean ± SD[range])	28.59 ± 35.97[8–160]	17.63 ± 22.88[8–121]	0.129610565
PCT (ng/ml, mean ± SD[range])	10.68 ± 23.74[0.07–100]	0.32 ± 0.27[0.08–1.08]	0.073222128
IL-6 (pg/ml, mean ± SD[range])	491.44 ± 1270.84[2.62–5000]	108 ± 157.73[5.89–489.6]	0.267581553
MRI	abnormal(22),normal(15)	abnormal(6),normal(12)	0.089123437
aEEG	abnormal(23),normal(6)	abnormal(10),normal(5)	0.467569421

Abbreviations: aEEG, amplitude integrated electroencephalography; BW, birth weight; CRP, C-reactive protein; CSF, cerebrospinal fluid; GA, gestational age; LC-MS/MS, liquid chromatography-tandem mass spectrometry; MEN, meningoencephalitis; MRI, magnetic resonance imaging.

To discover markers of neonatal sepsis with meningoencephalitis, we used DESeq2, a moderated method for differential analysis based on shrinkage estimation for dispersions and fold changes [25], together with the Wilcoxon rank sum test to identify differentially abundant metabolites in the CSF samples of patients with neonatal sepsis with meningoencephalitis or without meningoencephalitis. A total of 13 metabolites with significant differences between the two groups were detected; 9 metabolites were significantly increased in the group with meningoencephalitis, namely, pyridoxal, kynurenic acid, homovanillic acid, pyrrolidine, pyruvic acid, L-proline, dopamine, phenolglyoxylic acid, and glycocholic acid, and 4 metabolites were significantly decreased in the group with meningoencephalitis, namely, homo-l-arginine, urea, phosphoric acid, and creatinine, compared with their abundances in the group without meningoencephalitis. Similarly, the differential analysis of the metabolites in the serum of patients with neonatal sepsis with meningoencephalitis and without meningoencephalitis revealed 23 metabolites with significant differences between the two groups; 9 metabolites were significantly increased in the group with meningoencephalitis, namely, taurine, glycocholic acid, Italian acid, arabinosylhypoxanthine, hippuric acid, L-proline, pregnanetriol, betaine, and palmitic acid, and 14 metabolites were significantly decreased in the group with meningoencephalitis compared with neonatal sepsis without meningoencephalitis, namely, 2-methoxyacetaminophen sulfate, stearoylcarnitine, uric acid, creatine, 2-phenyl-4-pentenal, L-palmitoylcarnitine, 11-beta-hydroxyandrosterone-3-glucuronide, creatinine, 3-hydroxy-5, 8-tetradecadiencarnitine, indoxyl sulfate, androstenedione, homo-l-arginine, N1, N12-diacetylspermine, hexadecanedioic acid, and mono-L-carnitine ester, compared with their abundances in the group without meningoencephalitis. Notably, L-proline and glycocholic acid were increased significantly and homo-l-arginine and creatinine were decreased in the CSF and serum samples of the group with meningoencephalitis compared with their abundances in the group without meningoencephalitis.

To understand the biological roles of the different metabolites, we conducted a metabolite pathway enrichment analysis and found that the urea cycle, vitamin B6 metabolism, arginine and proline metabolism, glycolysis, and cysteine metabolism pathways were significantly enriched in the CSF samples of the group with meningoencephalitis compared with their enrichment in the group without meningoencephalitis (Supplementary Fig. 2a). Interestingly, the urea cycle and arginine and proline metabolism pathways, which both involve phosphoric acid, were decreased significantly in both the CSF and serum samples of the group with meningoencephalitis. Five pathways were significantly enriched in serum samples (Supplementary Fig. 2b) as follows: 1) in the creatine deficiency guanidinoacetate methyltransferase deficiency pathway, which involves creatine, uric acid, and creatinine, the relative content of these three metabolites was lower in the serum of the group with meningoencephalitis compared with the group without meningoencephalitis; 2) in the celiac disease pathway, which involves glycocholic acid, L-palmitoylcarnitine, and stearoylcarnitine, the relative content of glycocholic acid was higher and the relative content of L-palmitoylcarnitine and stearoylcarnitine was lower in the serum of the group with meningoencephalitis compared with the group without meningoencephalitis; 3) in the argininemia, hyperargininemia, arginase deficiency pathways, which involve creatine and homo-l-arginine, the relative content of these two metabolites was higher in the serum of the group with meningoencephalitis compared with the group without meningoencephalitis; 4) in the critical illness (major trauma, severe septic shock, or cardiogenic shock) pathway, which involves creatine and uric acid, the relative content of these two metabolites was higher in the serum of the group with meningoencephalitis compared with the group without meningoencephalitis; and 5) in the methylmalonic aciduria (MMA) pathway, which involves glycocholic acid, L-palmitoylcarnitine and stearoylcarnitine, the relative content of glycocholic acid was higher and the relative content of L-palmitoylcarnitine and stearoylcarnitine was lower in the serum of the group with meningoencephalitis compared with the group without meningoencephalitis.

Machine learning methods have been used successfully to find disease-related metabolite markers. Considering that it is relatively easier to obtain serum samples from patients with neonatal sepsis than CSF samples that require lumbar puncture, in this study, we chose to use serum samples to detect serum metabolic markers that would have more diagnostic potential in clinical settings than CSF markers. We applied machine learning methods to predict the concentration of CSF metabolite markers on the basis of the concentration of serum metabolite markers and compared the results with the identified meningoencephalitis-related markers. We compared the prediction results of different linear models and found that the lasso regression model performed best. Among the metabolite markers, the homo-l-arginine concentration predicted in the CSF by the lasso model on the basis of its concentration in the serum sample was the closest to the true CSF concentration (R^2^ = 0.811). The elastic net regression model gave the next best prediction for the CSF concentration of homo-l-arginine, whereas the predictions of the linear regression and ridge regression models were not good (R^2^ = 0.260 and 0.248, respectively) (Fig. 2**b**).

The lasso algorithm directly sets the regression coefficient with a small absolute value to 0 by constructing a penalty function, so that a more refined regression model can be obtained. This method is particularly suitable for reducing the number of features and selecting important features. We evaluated the serum metabolite markers that contribute to the target CSF metabolite concentration using the lasso model. We found that the serum concentrations of homo-l-arginine, hexadecanedioic acid mono-L-carnitine ester, and uric acid were positively correlated with the CSF concentration of homo-l-arginine (Fig. 2**c**).

The XGBoost algorithm is a scalable machine learning method based on tree boosting that has been applied successfully in omics [26], [27], [28]. We combined XGBoost and lasso to predict the composition of metabolites in CSF, and found that the CSF concentration of homo-l-arginine predicted on the basis of its concentration in the serum was significantly positively correlated with the actual serum concentration (Fig. 2**d**).

**Fig. 3:**
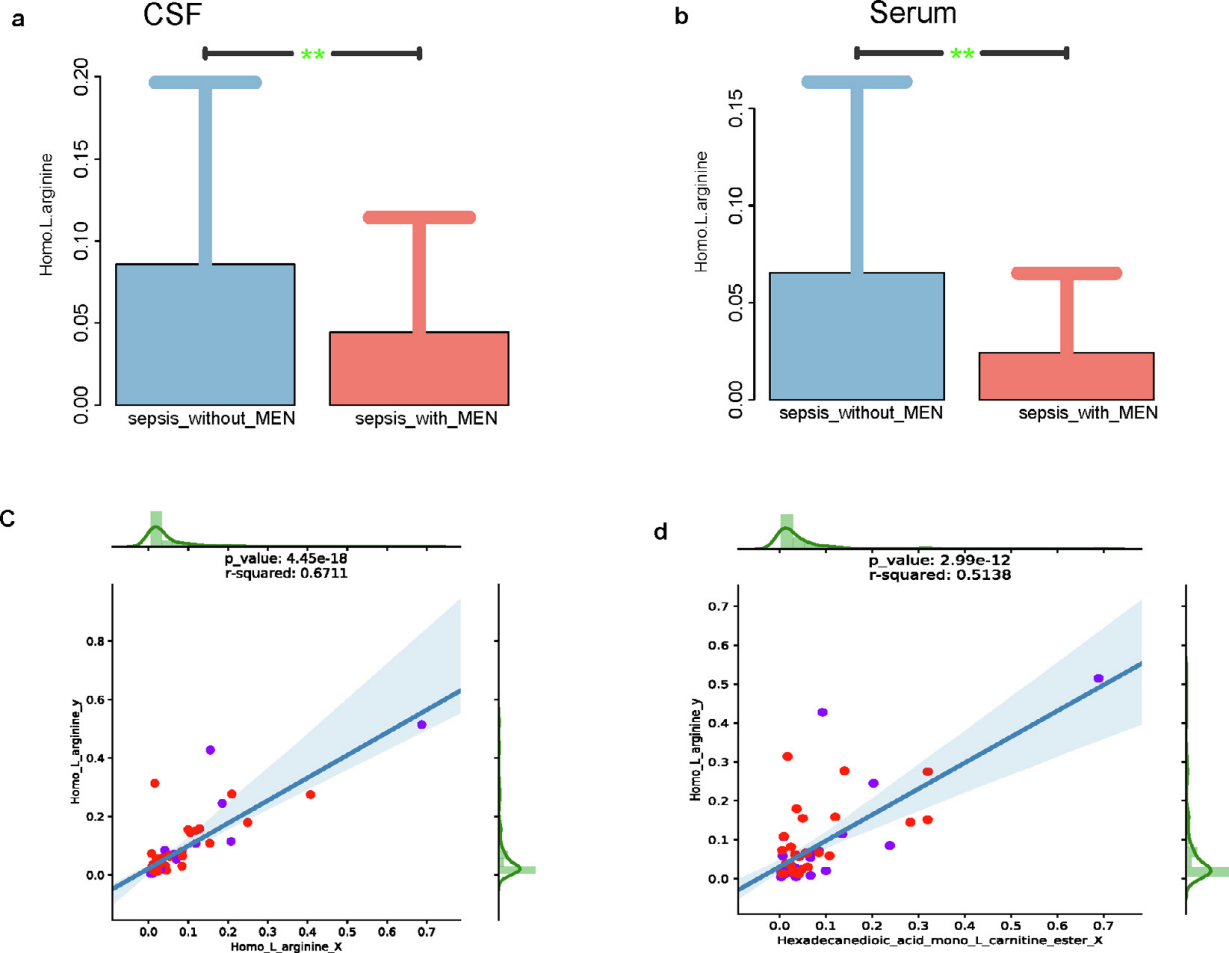
Homo-l-arginine concentrations in serum and cerebrospinal fluid (CSF) of neonatal sepsis with meningoencephalitis and without meningoencephalitis. (a, b) Homo-l-arginine concentrations in the CSF and serum of neonatal sepsis with meningoencephalitis were significantly reduced. (c, d) Homo-l-arginine concentration in the CSF of neonates with sepsis was significantly positively correlated with homo-l-arginine and hexadecanedioic acid mono-L-carnitine ester in the serum.

Together, these results confirmed that the concentrations of homo-l-arginine in the CSF and serum samples were significantly lower in the group with meningoencephalitis compared with the group without meningoencephalitis (Fig. 3**a, b**), and that the concentration of homo-l-arginine in the CSF of neonates with meningoencephalitis was significantly positively correlated with the concentrations of homo-l-arginine and hexadecanedioic acid mono-L-carnitine ester in the serum (Fig. 3**c, d**).

**Fig. 4:**
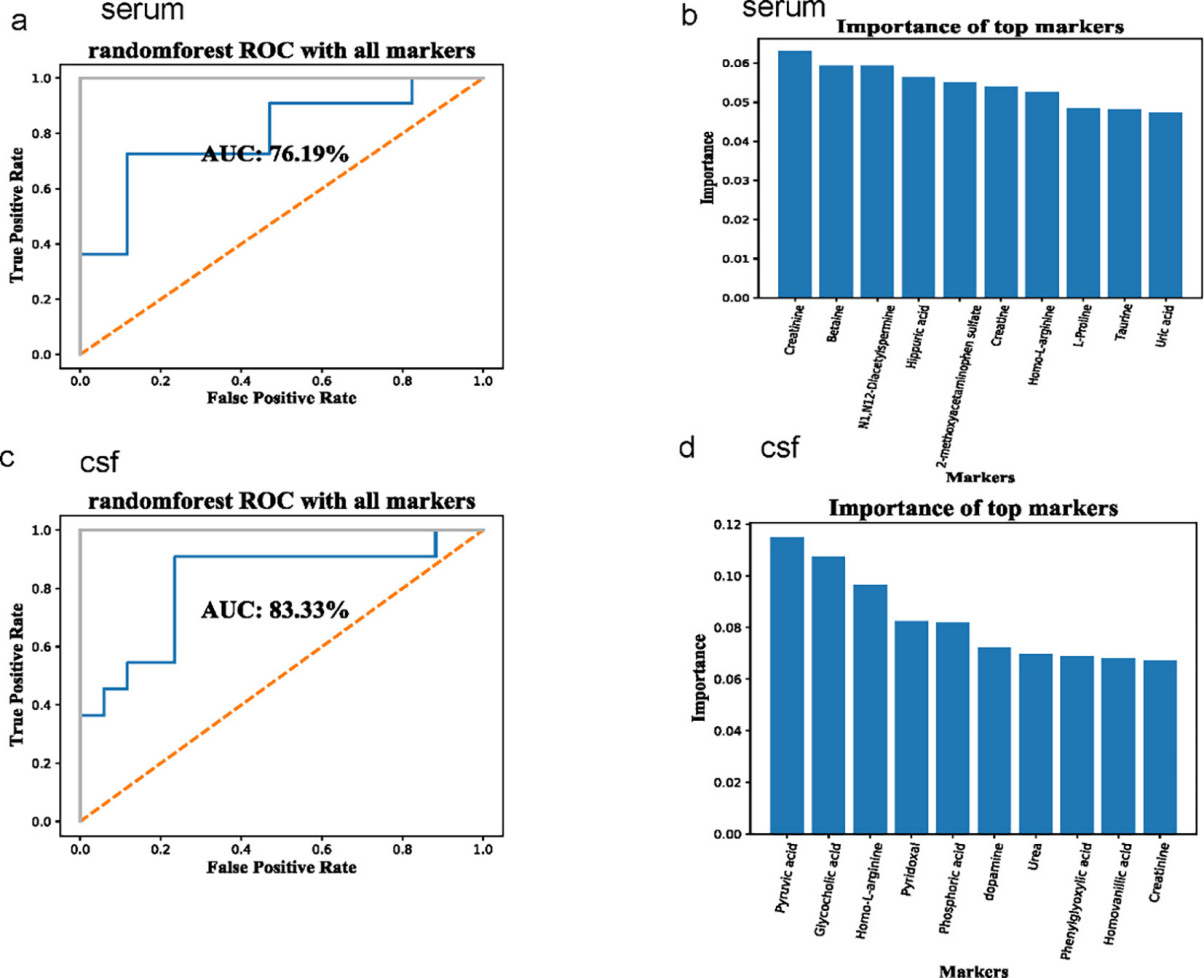
Metabolites are markers of neonatal sepsis with meningoencephalitis. (a, c) AUC values for serum and cerebrospinal fluid (CSF). (b, d) Importance of metabolite markers in serum and CSF determined using the Random Forest classifier model.

To further evaluate the clinical diagnostic value of the differentially abundant metabolites, we analyzed the ROC curves of these metabolites and found that the AUC was 76.19% for the serum metabolite markers (Fig. 4**a**), and that creatinine was ranked highest and homo-l-arginine was ranked in the top 10 by the Random Forest classifier model (Fig. 4**b**). The AUC was higher (83.33%) for the CSF metabolite markers (Fig. 4**c**), and pyruvic acid was ranked first and homo-l-arginine was ranked third by the Random Forest classifier model (Fig. 4**d**).

Together, these results indicate that the four differentially abundant metabolites are potential biomarkers for distinguishing meningoencephalitis in patients with neonatal sepsis.

## 4 Discussion

### 4.1 Alterations in arginine metabolism suggest abnormal nitric oxide synthesis in patients with neonatal sepsis with meningoencephalitis

Arginine is a semi-essential amino acid for healthy adults, but is essential for premature babies, newborns, or severely ill patients. Previous studies established the correlation between sepsis and plasma arginine concentrations. Argaman *et al.* found that the plasma arginine concentration was significantly reduced in severely ill children [30]. Lee *et al.* found that supplementation of l-arginine and L-citrulline improved the function of regulatory T cells and improved the prognosis of sepsis in model mice [30]. Yeh *et al.* found that when sepsis model mice were injected intravenously with arginine, the mobilization of circulating angiogenic cells was enhanced, homeostasis of the angiopoietin (Angpt)/Tie-2 axis was maintained, and distal organ damage of multiple sepsis was reduced [31]. Together, these studies indicated that arginine deficiency may result in unfavorable outcomes of sepsis. Consistent with these studies, we found that the concentrations of homo-l-arginine in both the CSF and serum samples of the group with meningoencephalitis were significantly reduced compared with the concentrations in the group without meningoencephalitis. We also found the changes in arginine metabolism in the serum and CSF were significantly related.

Homo-l-arginine is a non-protein amino acid and an arginine derivative. Arginine and homo-l-arginine are both direct precursors of nitric oxide (NO) synthesis. NO, as a gas messenger produced by the enzymatic activity of nitric oxide synthase, was identified as an important factor in vascular dysfunction in sepsis [32], [33], [34]. Low serum levels of homo-l-arginine has been reported as a risk factor for cardiovascular disease in adults [35], [36], and targeting NO synthesis is a potential treatment for sepsis [37]. However, little is known about the importance of the l-arginine/homo-l-arginine/nitric oxide (Arg/hArg/NO) pathway in children, especially its role in neonatal sepsis. Buck *et al.* investigated the Arg/hArg/NO pathway in 106 preterm infants and found that homo-l-arginine biosynthesis in preterm infants was positively correlated with gestational age, suggesting that homo-l-arginine may be involved in fetal growth [38]. McDonald *et al.* found that poor delivery outcomes caused by malaria infection during pregnancy were related to lower concentrations of arginine and higher concentrations of endogenous inhibitors of NO biosynthesis, and, using animal models, they found that supplementing l-arginine improved birth outcomes by normalizing the angiogenesis pathway and enhancing placental vascular development [39].

NO has been reported to be involved in neuronal signal transduction and inflammation. Boyko *et al.* found that the NO produced by Arg and homo-l-arginine in the cortex of rats with severe spinal cord injury was significantly reduced [40]. The changes in arginine metabolism that we found in the serum and CSF samples in the group with meningoencephalitis together with the results of the previous studies confirm the important role of the Arg/hArg/NO pathway in neonatal sepsis.

### 4.2 Alterations in creatinine metabolism in neonatal sepsis with meningoencephalitis suggest imbalance of energy homeostasis linked to arginine metabolism

Serum creatinine is a marker of acute kidney injury in critically ill neonates [41]. Legrand *et al.* found that sepsis can reduce creatinine production [42], but the specific role of changes in serum creatinine levels in the pathogenesis of sepsis is still unclear. Considering that arginine can be converted directly to homo-l-arginine and guanidinoacetic acid (GAA) by arginine:glycine amidinotransferase, a decrease in the homo-l-arginine level may be accompanied by a decrease in the GAA level. Considering that GAA is the direct precursor of creatine, we consider that changes in creatine metabolism may be related to changes in arginine metabolism.

Changes in energy homeostasis have been shown to lead to sepsis-mediated multiple organ failure, and creatine is important in maintaining energy balance [5]. Creatine is synthesized in the liver by GAA through S-adenosylmethionine methylation, and is essential for muscle activity. Phosphokinase catalyzes creatine to form high-energy creatine phosphate, and creatine phosphate is hydrolyzed to release energy and creatinine and phosphoric acid when energy is needed by the body [43]. We found that the serum phosphoric acid concentration was significantly lower in the group with meningoencephalitis compared with its concentration in the group without meningoencephalitis, which suggests the presence of energy homeostasis in neonatal sepsis with meningoencephalitis.

### 4.3 Changes in oxidative stress-related markers and potentially harmful bile acid and aromatic compounds in neonatal sepsis with meningoencephalitis

Oxidative stress and the production of intracellular reactive oxygen species are related to the pathogenesis of sepsis. Xu *et al.* found that exogenous and endogenous antioxidants, ascorbic acid, taurine, and glutathione had beneficial effects on septic rats by protecting mitochondria [44]. Consistent with this finding, we found that the concentration of antioxidant taurine in the serum of the group with meningoencephalitis was significantly increased compared with its concentration in the group without meningoencephalitis. Proline is considered to be an effective antioxidant, and a proline–arginine-rich host defense peptide was shown to have efficacy in rodent bacteremia models [44], [45]. Consistent with the findings of these studies, we found that the proline concentration was significantly increased in both the serum and CSF samples of the group with meningoencephalitis compared with its concentration in the group without meningoencephalitis.

Bile acids, which control inflammation by interacting with several receptors, have been reported to play important roles in the pathogenesis of sepsis [46], [47]. We found that the concentration of glycocholic acid, a cytotoxic bile acid derivative, was significantly increased in the group with meningoencephalitis compared with its concentration in the group without meningoencephalitis.

Abnormal metabolism of aromatic compounds is considered a potential clinical indicator of sepsis [48]. Bhuiyan *et al.* found that *Acinetobacter baumannii* phenylacetic acid metabolism directly affected the outcome of the infection by regulating the chemotaxis of neutrophils [49]. Consistent with this finding, we found that the concentration of phenylglyoxylic acid, an aromatic compound involved in phenylacetic acid metabolism pathway, was significantly increased in the CSF of the group with meningoencephalitis compared with its concentration in the group without meningoencephalitis. We also found that the concentration of the aromatic compound hippuric acid was significantly decreased in the group with meningoencephalitis compared with its concentration in the group without meningoencephalitis. Together, these results suggest that the metabolism of aromatic compounds was different in the groups with meningoencephalitis and septic patients without meningoencephalitis.

**Fig. 5:**
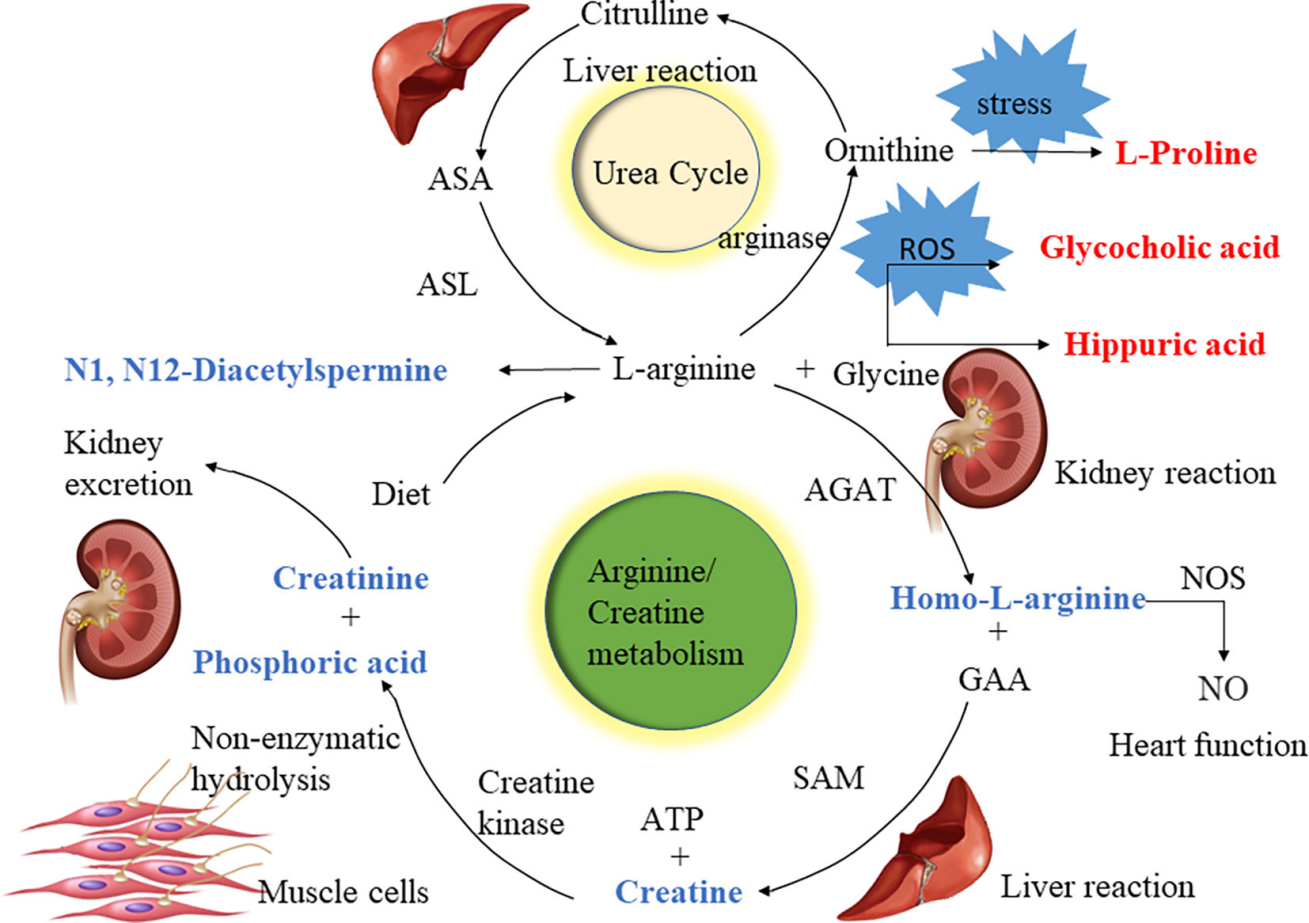
Central role of arginine metabolism in changes in the cerebrospinal fluid and serum metabolomes of neonatal sepsis with meningoencephalitis. Changes in arginine metabolism were closely related to changes in creatinine metabolism, as well as changes in oxidative stress-related markers and potentially harmful bile acid and aromatic compound metabolism. Metabolites in red/blue were enriched/decreased in neonatal sepsis with meningoencephalitis. (For interpretation of the references to colour in this figure legend, the reader is referred to the web version of this article.) (For interpretation of the references to colour in this figure legend, the reader is referred to the web version of this article.)

In summary, we speculate that the changes in the CSF and serum metabolomes of the group with meningoencephalitis were manifested mainly as changes in arginine metabolism, which were closely related to changes in creatinine metabolism, oxidative stress-related markers, and potentially harmful bile acid and aromatic compound metabolism, as illustrated in Fig. 5.

### 4.4 Innovation and limitations

We describe a novel approach in which the CSF and serum samples were collected from each patient at the same time, non-targeted metabolomics testing was performed, and machine learning methods were used to screen neonatal sepsis markers related to meningoencephalitis. However, due to the retrospective study, it was often prone for bias, and not every participants finished the EEG or MRI examination which could help to identify neonatal sepsis with meningoencephalitis. Because of differences in the environments of serum and CSF, there were limitations in finding common different metabolites in the two environments. The machine learning method LASSO performed well in predicting the concentrations of metabolites in CSF on the basis of serum metabolite levels. Whether neonatal sepsis with meningoencephalitis can be predicted on the basis of serum metabolite concentrations needs to be further investigated.

## 5 Conclusions

Analysis of the serum and cerebrospinal fluid metabolomes combined with machine learning identified metabolite markers related to neonatal sepsis with meningoencephalitis. The characteristics of neonatal sepsis meningoencephalitis-were manifested mainly by changes in arginine metabolism and related changes in creatinine metabolism.

## Funding

This work was supported by grants from the 10.13039/501100001809National Natural Science Foundation of China (Program Nos. 82071733), the 10.13039/501100012166National Key Research and Development Program of China (No. 2017YFA0104204), Shanghai talent development funding (No. 2020115), by Shenzhen Science Technology and Innovation Commission (JCYJ20190807152403624), by Longgang Science Technology and Innovation Commission of Shenzhen (LGKCYLWS2018000048), and by High Level Project of Medicine in Longhua, ShenZhen(No. HLPM201907020103).
