# Supplementary material for: Machine learning applied to serum and cerebrospinal fluid metabolomes revealed altered arginine metabolism in neonatal sepsis with meningoencephalitis
Source: Comput Struct Biotechnol J. 2021 May 18;19:3284–92. doi: 10.1016/j.csbj.2021.05.024 (PMC8207169; doi:10.1016/j.csbj.2021.05.024)
Supplement: Supplementary data 1 [file mmc1.docx]

**Supplementary Figures**


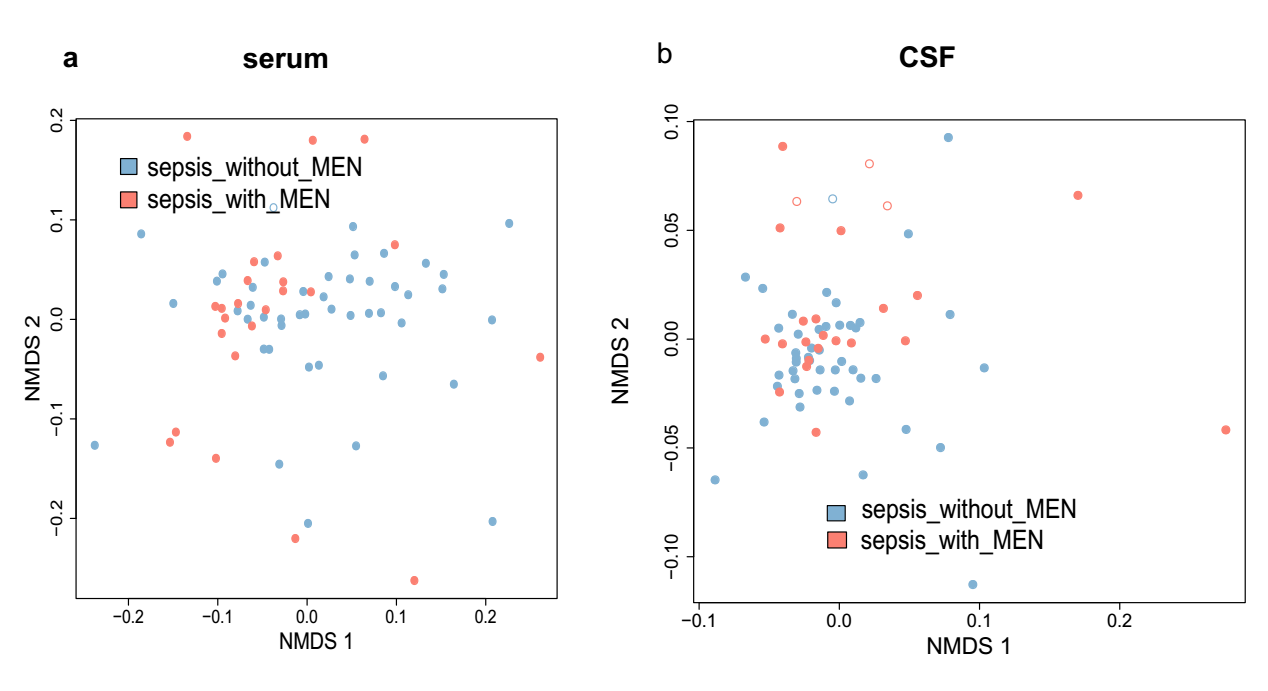


**Supplementary Figure 1.** **Non-metric multidimensional scaling (NMDS) analysis of the cerebrospinal fluid (CSF) (a) and serum (b) samples.** MEN, meningoencephalitis.

**
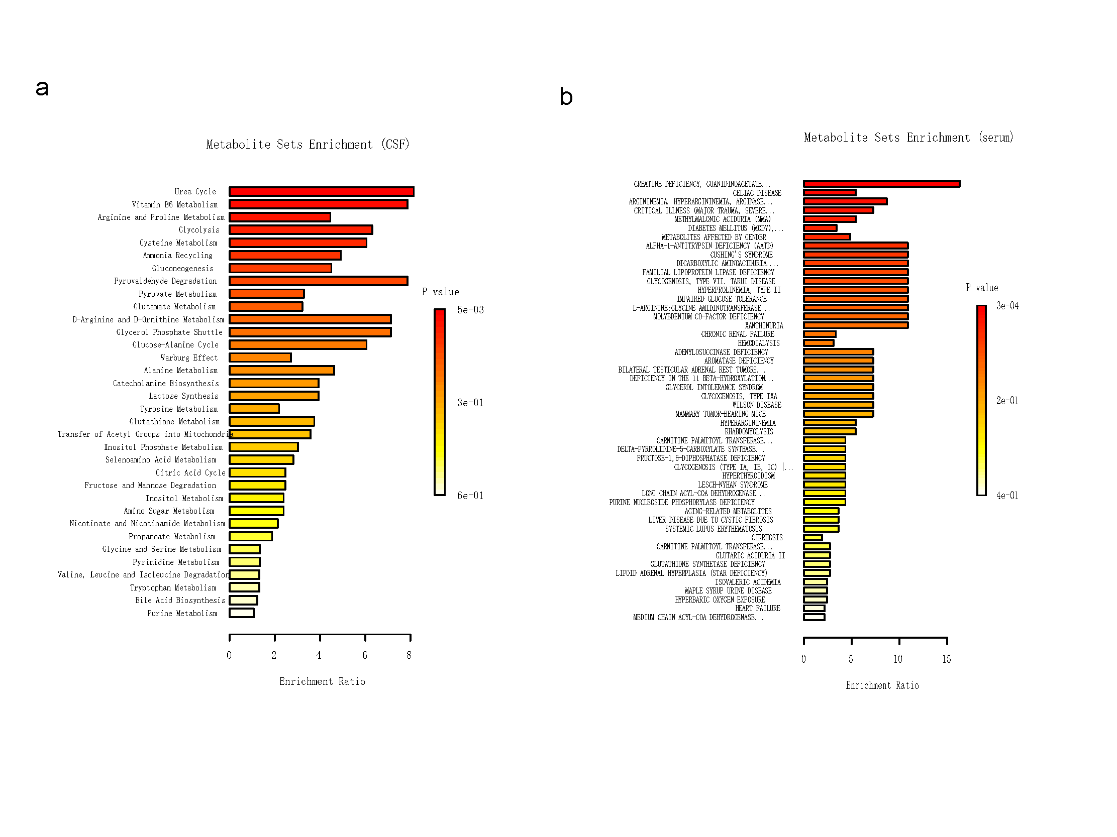
**

**Supplementary Figure 2.** **Metabolite pathways enrichment analysis of the cerebrospinal fluid (CSF) (a) and serum (b) samples.**
